# The Clinical Significance and Therapeutic Management of Dactylitis and Enthesitis in Psoriatic Arthritis

**DOI:** 10.31138/mjr.210725.ewr

**Published:** 2026-01-08

**Authors:** Eleftherios Pelechas, Panagiota G. Karagianni, Evripidis Kaltsonoudis

**Affiliations:** 1 Chatzikosta General Hospital, Department of Rheumatology, Ioannina, Greece;; 2 University of Ioannina, Medical School, Department of Microbiology, Ioannina Greece

**Keywords:** psoriatic arthritis, dactylitis, enthesitis, biologics, TNF inhibitors, personalised medicine

## Abstract

**Introduction:**

Dactylitis and enthesitis are hallmark musculoskeletal manifestations of psoriatic arthritis, often signifying a more severe disease phenotype. These domains not only aid early diagnosis but are also associated with radiographic progression, functional impairment, and reduced quality of life.

**Objective:**

This review aims to synthesise current insights into the pathophysiology, clinical impact, imaging assessment, and targeted therapeutic strategies for dactylitis and enthesitis in PsA, within the framework of precision medicine.

**Methods:**

A comprehensive analysis of the literature was conducted, focusing on the immunobiology, imaging modalities (ultrasound, MRI), validated clinical scoring systems, and the efficacy of conventional, biologic, and targeted synthetic DMARDs in the treatment of dactylitis and enthesitis.

**Results:**

Both manifestations arise from inflammation at the bone-entheseal interface, mediated by biomechanical triggers and the IL-23/IL-17 axis. Dactylitis involves tenosynovitis, soft tissue oedema, and synovitis, while enthesitis reflects inflammation at tendon and ligament insertions. Imaging enhances diagnostic accuracy and monitors subclinical disease. TNF and IL-17 inhibitors demonstrate high efficacy in these domains, whereas IL-23 and JAK inhibitors offer promising alternatives. Personalised treatment algorithms now integrate domain predominance, prognostic markers, comorbidities, and patient preferences.

**Conclusion:**

Dactylitis and enthesitis represent pivotal, prognostically relevant domains in PsA. Their early recognition and domain-specific management are critical to optimising outcomes. Advances in imaging, novel therapeutic targets, and biomarker-driven strategies hold promise for more effective, individualised care.

## INTRODUCTION

Psoriatic arthritis (PsA) is a chronic, immune-mediated inflammatory musculoskeletal disorder that affects approximately 20–30% of individuals with psoriasis.^1^ It is classified within the spectrum of spondyloarthritides (SpA) and is characterised by a diverse range of clinical manifestations.^2^ The disease course is highly heterogeneous, with patients experiencing varying degrees of peripheral arthritis, axial involvement, dactylitis, enthesitis, and extra-articular features such as skin and nail psoriasis.^3^ The variability in clinical presentation poses challenges in diagnosis and management, particularly in early disease stages. Among the hallmark features of PsA, dactylitis referred to as “sausage digit” and enthesitis are considered key clinical domains that help distinguish PsA from other inflammatory arthritides such as rheumatoid arthritis (RA) and ankylosing spondylitis (AS). These manifestations are not only distinctive but are also associated with a more aggressive disease phenotype, greater functional impairment, and reduced quality of life.^4^

From a pathophysiological standpoint, both dactylitis and enthesitis reflect the central role of inflammation at the bone-entheseal interface, a key feature in PsA distinct from synovial-predominant inflammation seen in RA.^5^ These manifestations are thought to arise from biomechanical stress, genetic predisposition (e.g. HLA-B27), and dysregulated innate and adaptive immune responses, including overexpression of cytokines such as TNF-α, IL-17, and IL-23.^6^

Given their clinical relevance, the accurate assessment and effective management of dactylitis and enthesitis are critical components in optimising patient outcomes in PsA. Modern treatment strategies increasingly focus on targeting these domains, and several biologic and targeted synthetic (ts) disease-modifying anti-rheumatic drugs (DMARDs) have shown varying degrees of efficacy. Therefore, understanding the therapeutic responses of these key disease manifestations is essential for tailoring individualised treatment plans and improving long-term disease control.^7^

A comprehensive literature search was conducted to identify relevant studies on the clinical significance, assessment, and therapeutic management of dactylitis and enthesitis in psoriatic arthritis.^8^ The search was performed in PubMed/MEDLINE up to July 2025. The following combination of Medical Subject Headings and free text terms was used: (“psoriatic arthritis” OR “psoriatic disease”) AND (“dactylitis” OR “sausage digit”) AND (“enthesitis” OR “enthesopathy”) AND (“treatment” OR “therapy” OR “management” OR “biologic” OR “targeted synthetic DMARD” OR “IL-17 inhibitors”). No language restrictions were applied, but most articles were in English. Conference abstracts were considered if they provided sufficient methodological detail.

This review has certain limitations that should be acknowledged. Although a comprehensive literature search was conducted, it was not a systematic review, and therefore some relevant studies may have been inadvertently omitted. The selection of articles was based on the authors’ judgment of clinical relevance. Additionally, the evidence available for certain therapeutic approaches to enthesitis and dactylitis in PsA remains limited, particularly in head-to-head comparative studies.

## PATHOPHYSIOLOGY

### Dactylitis

**Figure 1.**
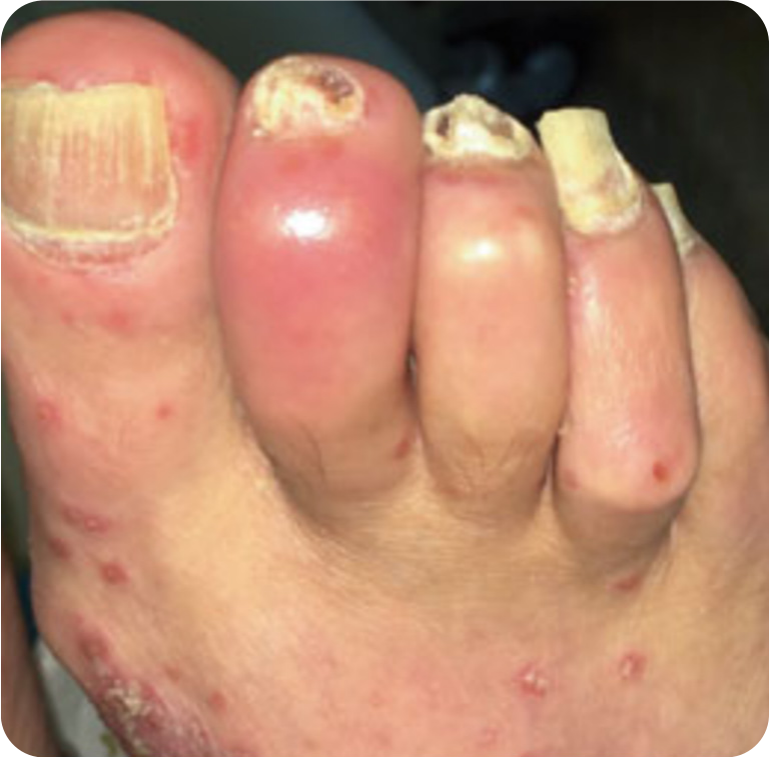
Typical presentation of acute dactylitis in a patient with PsA *(Pelechas et al. Illustrated Handbook of Rheumatic and Musculoskeletal Diseases. Springer)*

Dactylitis, colloquially known as “sausage digit”, represents a hallmark feature of PsA and is characterised by diffuse, fusiform swelling of an entire digit, typically a finger or toe (**Figure 1**). Unlike localised joint inflammation, dactylitis reflects a more extensive inflammatory process involving several anatomical compartments, including the synovium, flexor tendon sheaths (tenosynovitis), entheses, and surrounding soft tissues. This multifocal inflammation accounts for the characteristic uniform swelling and is often accompanied by erythema, warmth, and varying degrees of tenderness.^9^ From a pathological standpoint, both acute and chronic forms of dactylitis have been described. In acute dactylitis, histopathological analysis demonstrates prominent neutrophilic infiltration, oedema, and inflammatory changes in the tenosynovial tissue. In contrast, chronic dactylitis is associated with fibroblast proliferation, fibrosis, neoangiogenesis, and persistent low-grade inflammation.^10^ Imaging modalities such as musculoskeletal ultrasound (MSUS) and MRI further corroborate these findings by showing flexor tenosynovitis, joint synovitis, soft tissue oedema, and bone marrow oedema in affected digits.^11^

Recent insights into the immunopathogenesis of dactylitis underscore the central role of innate immunity and the IL-23/IL-17 axis. The flexor tendon apparatus, particularly at the level of the digit, is susceptible to biomechanical stress and microtrauma, which may act as a trigger for local immune activation. This process involves resident mesenchymal cells and innate lymphoid cells that respond to cytokines such as IL-23, producing effector cytokines including IL-17A and TNF-α, which in turn drive local inflammation and tissue remodelling.^12^

### Enthesitis

**Figure 2.**
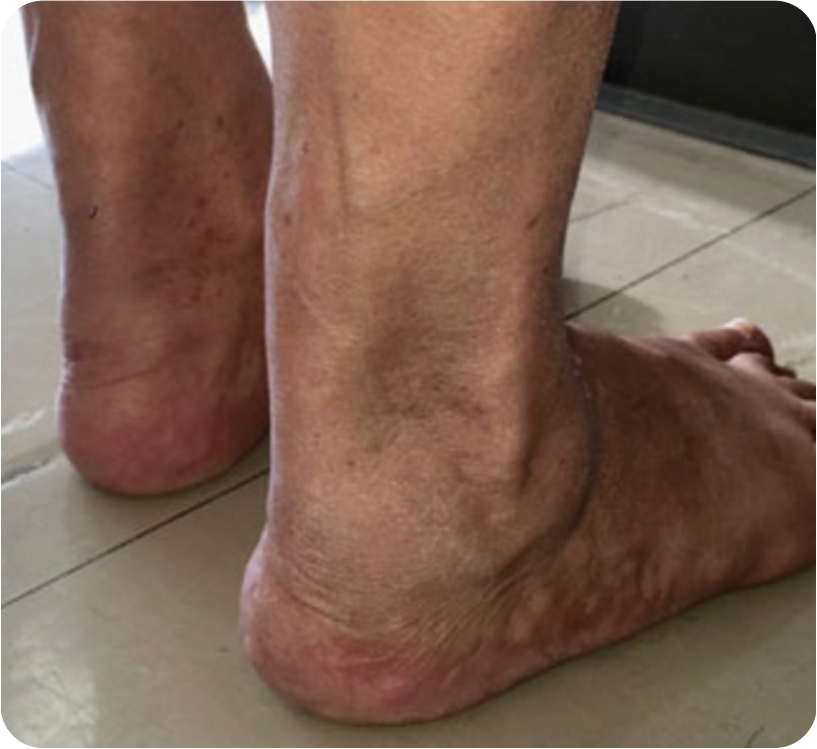
Achilles tendon involvement occurs in a significant number of patients. In this case, swelling of the right Achilles tendon is shown. *(Pelechas et al. Illustrated Handbook of Rheumatic and Musculoskeletal Diseases. Springer)*

Enthesitis is a defining pathophysiological feature of PSA and other SpAs. It often manifests clinically as pain, tenderness, and functional limitation at sites of high mechanical load such as the Achilles tendon (**Figure 2**), plantar fascia, and the lateral epicondyle. Unlike isolated tendinopathies, enthesitis in PsA represents a complex immunological phaenomenon rather than a purely mechanical disorder.^13^

Traditionally considered a biomechanical interface, the enthesis is now recognised as a unique immunological niche, populated by resident immune cells including macrophages, dendritic cells, γδ T cells, and innate lymphoid cells.^14^ Mechanical stress and microdamage can disrupt the local barrier and initiate inflammation by releasing damage-associated molecular patterns (DAMPs), which in turn activate pattern recognition receptors (PRRs) on resident cells. This cascade culminates in the production of pro-inflammatory cytokines, prominently IL-23, which drives downstream expression of IL-17 and IL-22, key cytokines implicated in PsA pathogenesis.^15^

Imaging studies using MSUS and MRI frequently reveal entheseal thickening, increased vascularity, erosions, and bone proliferation, supporting the notion that enthesitis is not merely soft tissue inflammation but also involves adjacent bone and periosteum. Importantly, enthesitis can precede synovitis and is thought to be a primary lesion in the disease process of PsA, further highlighting its clinical therapeutic relevance.^16^

## CLINICAL SIGNIFICANCE

### Diagnostic value

Dactylitis and enthesitis are key clinical domains in the diagnosis and classification of PsA, particularly in early or oligoarticular forms where classic features such as symmetrical polyarthritis may be absent. These manifestations are often among the earliest signs of PsA and can precede the development of overt synovitis. The CASPAR (Classification criteria for Psoriatic Arthritis) criteria explicitly incorporate both dactylitis and enthesitis as important clinical features. The presence of current dactylitis, or a history of dactylitis recorded by a rheumatologist, contributes to the classification score. Similarly, tender entheseal points, particularly at characteristic sites such as the Achilles tendon or plan-tar fascia, can support the clinical suspicion of PsA.^17^

Advanced imaging modalities have further improved the diagnostic utility of these features. MSUS^18^ and MRI^19^ can detect subclinical enthesitis and dactylitis, offering valuable insight in patients with equivocal symptoms or in cases where traditional inflammatory markers are within normal limits. Their identification may prompt earlier referral to a rheumatologist and initiation of targeted therapy.

### Prognostic implications

The presence of dactylitis and enthesitis is not diagnostically relevant but also associated with more aggressive disease phenotypes. Dactylitis has been consistently linked to radiographic damage, particularly erosions in small joints of the hands and feet. Early-onset dactylitis is predictive of a higher burden of joint destruction, underscoring the need for timely therapeutic intervention. In cohort studies, patients with recurrent or persistent dactylitis often demonstrate greater long-term structural damage compared to those without.^20^

Enthesitis, on the other hand, is correlated with higher composite disease activity indices, such as the DAPSA (Disease Activity in Psoriatic arthritis) score, as well as with extra-articular symptoms like fatigue and sleep disturbance. Enthesitis may signal a more inflammatory systemic phenotype and has been associated with poorer physical functioning and higher health care utilisation. Moreover, the presence of multiple entheseal sites tends to predict a refractory disease course and may be indicative of the need for biologic therapy.^21^

### Impact on quality of life

From the patient’s perspective, both dactylitis and enthesitis have a substantial impact on daily function and well-being. Dactylitis, particularly when affecting dominant digits, interferes with fine motor skills, grip strength, and manual tasks, while enthesitis contributes to persistent pain at rest and during movement, often mimicking mechanical pain and leading to diagnostic delays.^22^

Notably, pain form enthesitis is frequently under-recognised yet may be perceived by patients as more distressing and debilitating than joint pain. It is often diffuse and poorly localised, which contributes to mis-diagnosis as fibromyalgia or non-inflammatory conditions. Patient-reported outcomes such as the PsAID (Psoriatic Arthritis Impact of Disease) questionnaire consistently reflect a disproportionate burden of symptoms in those with active enthesitis or dactylitis.^23^

The cumulative effect of these domains results in decreased physical activity, reduced work productivity, and impaired mental health, reinforcing the importance of routine assessment and comprehensive management strategies targeting these features specifically.

## IMAGING MODALITIES

### Dactylitis

**Figure 3.**
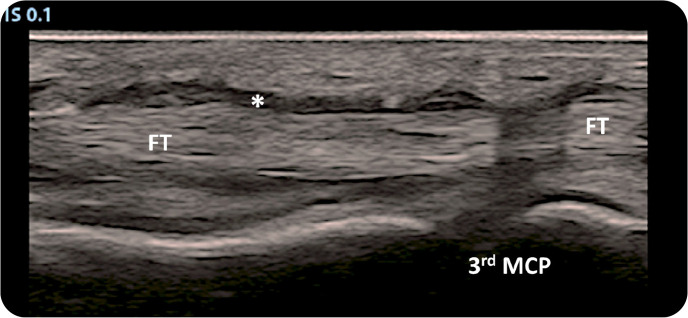
Longitudinal grayscale musculoskeletal ultrasound of the 3^rd^ finger. The flexor tendon appears thickened with hypoechogenicity around due to fluid accumulation (*) within the tendon sheath, suggestive of active tenosynovitis. The tendon sheath is slightly distended. There is also evidence of increased echogenicity and mild swelling of the surrounding soft tissues, indicating subclinical soft tissue oedema commonly associated with dactylitis. FT**:** flexor tendon**;** MCP: metacarpophalangeal joint. *(Pelechas et al. Illustrated Handbook of Rheumatic and Musculoskeletal Diseases. Springer)*

**Figure 4.**
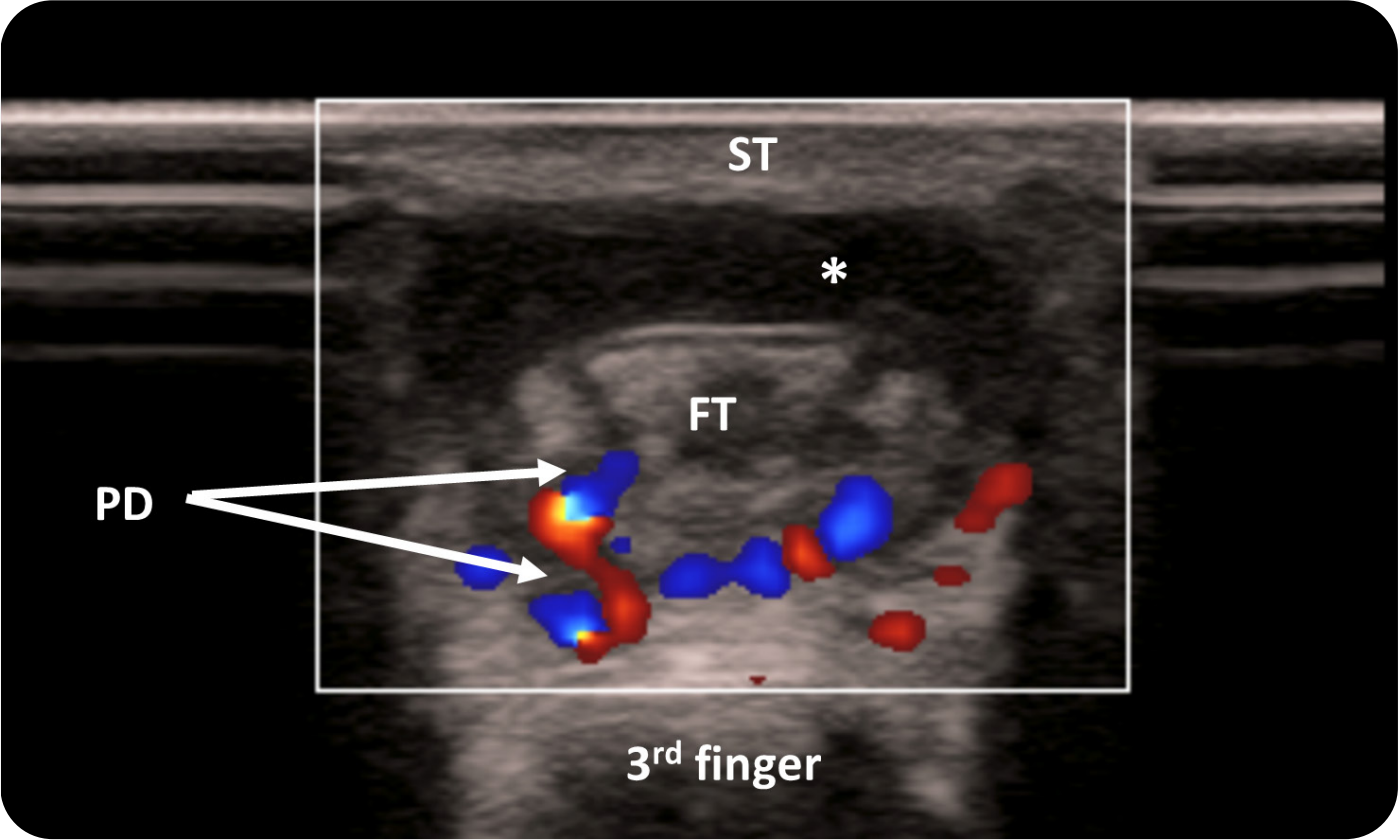
Transverse Power Doppler musculoskeletal ultrasound of the 3^rd^ finger. The flexor tendon is in cross-section and appears thickened and surrounded by hypoechoic fluid suggesting tenosynovial effusion (*). There is marked Doppler activity in the peritendinous region, indicating increased vascularisation, which is a hallmark of active inflammation. This is typically seen in psoriatic dactylitis. FT: flexor tendon; ST: soft tissue; PD: Power Doppler. *(Pelechas et al. Illustrated Handbook of Rheumatic and Musculoskeletal Diseases. Springer)*

Imaging plays a pivotal role in the assessment and characterisation of dactylitis in PsA. MSUS and MRI are the two most widely used modalities and provide complementary information that supports both diagnosis and disease monitoring. MSUS offers high spatial resolution and real-time visualisation of multiple anatomical structures involved in dactylitis. It is particularly effective in identifying: a) flexor tenosynovitis: often appearing as hypoechoic fluid around the flexor tendons with or without power Doppler (PD) signal indicating active inflammation; b) joint synovitis: characterised by synovial hypertrophy and effusion; c) subcutaneous tissue oedema: which may be seen as increased echogenicity in the surrounding soft tissues, reflecting inflammatory involvement beyond the synovium; d) peritendinous changes: especially around digital flexors (**Figures 3 and 4**). A recent article, explored the role of MSUS in toe dactylitis, which is a domain of limited application. Tenosynovitis and soft tissue oedema were the most common MSUS elementary lesions in acute toe dactylitis in PsA.^24^

The use of PD MSUS enhances the detection of vascularised inflammation and helps distinguish active from inactive disease. Its non-invasive nature, wide availability, and lack of radiation make MSUS an ideal tool for both routine clinical assessment and follow-up in patients with dactylitis.^25^

MRI, while less accessible, provides excellent soft tissue contrast and is particularly useful for: a) detecting bone marrow oedema: a key indicator of inflammation that often precedes structural damage; b) visualising deep tissue involvement: including tendon insertion points and joint capsule inflammation; c) identifying subclinical lesions: in early disease or in patients with equivocal clinical findings.

Together, these two modalities facilitate a more precise understanding of the extent and activity of dactylitis, enabling targeted therapeutic decisions.^26^

### Enthesitis

Imaging of enthesitis has undergone significant advances with the application of high-resolution ultrasound and whole-body MRI, improving diagnostic sensitivity and enabling the evaluation of subclinical disease.

**Figure 5.**
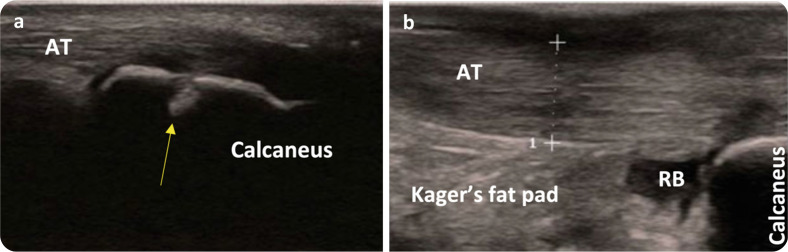
**(above).** Posterior longitudinal scan of the Achilles tendon. In 5A, a bone erosion is shown (yellow arrow) which is considered a relatively specific finding for PsA. In 5b, a thickened Achilles tendon with retrocalcaneal bursitis in a patient with PsA and heel pain. AT: Achilles tendon; RB: retrocalcaneal bursa. *(Pelechas et al. Illustrated Handbook of Rheumatic and Musculoskeletal Diseases. Springer)*

MSUS is currently the most practical and widely used modality in clinical rheumatology for the assessment of enthesitis. It allows for detailed visualisation of: a) *enthesophytes;* b) *calcifications*, both intra-tendinous and perientheseal; c) erosions (**Figure 5A**), which suggest chronic inflammatory damage; d) thickening of the tendon or ligament at the insertion site (**Figure 5B**); e) PD signal, indicating active entheseal inflammation.^27^

These findings can be quantified using validated scoring systems, the most prominent being:

- Madrid Sonographic Enthesitis Index (MASEI): assesses six bilateral entheseal sites and integrates both structural and inflammatory changes.^28^
- Glasgow Ultrasound Enthesitis Scoring System (GUESS): focuses on lower limb entheses and is useful for screening and follow-up.^29^

MSUS is especially advantageous for peripheral entheses, such as the Achilles tendon, plantar fascia, and quadriceps tendon. It enables serial evaluation to assess response to therapy and detect early signs of recurrence. Despite its utility, MSUS in the assessment of enthesitis has certain limitations. Interpretation can be subject to inter- and intra-observer variability, as image acquisition and scoring are operator-dependent. In addition, finding may be influenced by probe positioning, applied pressure, and patient-related factors, which can affect reproducibility and diagnostic consistency.

**Figure 6.**
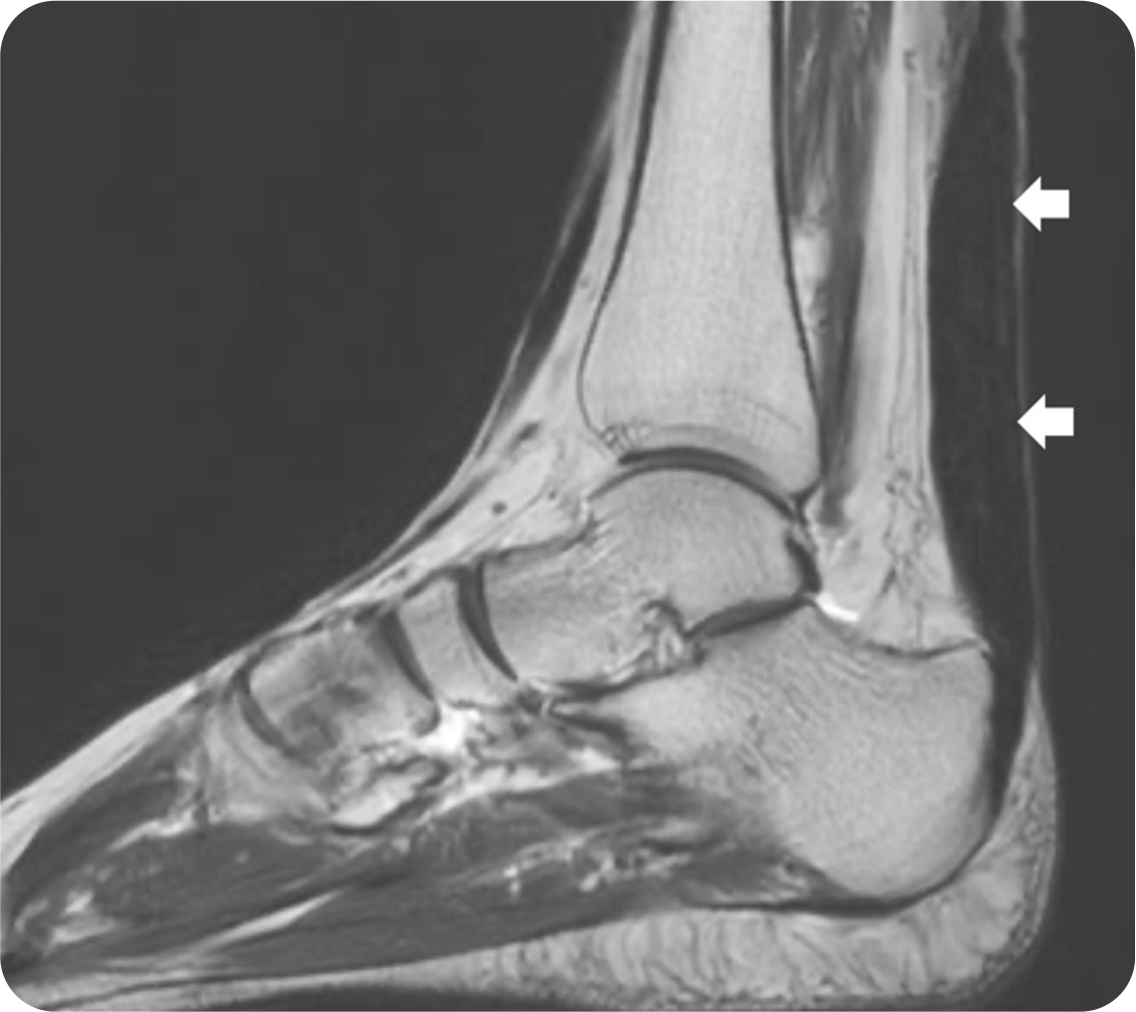
**(below).** MRI of the Achilles tendon. PD mDIXON image on MRI in sagittal plane revealed diffuse fusiform thickening (white arrows) in full length of the Achilles tendon in a PsA patient. *(Pelechas et al. Illustrated Handbook of Rheumatic and Musculoskeletal Diseases. Springer)*

On the other hand, MRI complements MSUS by allowing visualisation of the common tendons/entheses (**Figure 6**) but also of deep-seated entheses, particularly in axial sites like the spine, sacroiliac joints, and pelvis, which are not easily accessible by MSUS. MRI is also beneficial in paediatric and early PsA cases where subtle inflammatory changes may not yet be visible on conventional imaging. Importantly, both modalities have demonstrated the presence of subclinical enthesitis in patients with psoriasis alone, underscoring their potential for identifying individuals at risk for progression to PsA.

## ASSESSMENT TOOLS

### Dactylitis

The assessment of dactylitis in PsA encompasses both clinical observation and objective measurements. Accurate evaluation is important for disease monitoring, outcome assessment, and treatment response in both clinical practice and trials. Key approaches are shown in **Table 1.**

**Table 1.** Demographic and clinical characteristic of the patients.

**Approach**	**Description**
**Clinical examination**	Dactylitis is classified as tender or non-tender Inspection and palpation identify swelling, redness, and warmth to differentiate from other causes
**Digital circumference measurement**	involves measuring the circumference of affected digits and comparing with the contralateral side or normative values useful in monitoring progression or response to treatment
**Leeds Dactylitis Instrument (LDI)** **30**	a validated tool combining digit circumference and tenderness scoring LDI-b (basic): assesses tenderness only LDI-negativity: no swelling or tenderness (resolution) Ideal for clinical trials
**Imaging (MSUS/MRI)**	not part of formal scoring tools but can detect subclinical inflammation Useful in ambiguous clinical presentations or for additional confirmation

### Enthesitis

The assessment of enthesitis relies on palpation-based scoring systems and is increasingly supported by imaging tools. Given that enthesitis may be asymptomatic or overlap with fibromyalgia, accurate and reproducible evaluation is critical. Common clinical indices are shown in **Table 2.**

**Table 2.** Common clinical indices.

**Index**	**Description**
**Leeds Enthesitis Index (Lei)** **31**	Assesses tenderness at 6 sites: bilateral lateral epicondyles (elbows), medial femoral condyles (knees), and Achilles tendon insertions Simple and fast, ideal for routine clinical use
**Maastricht AS Enthesitis Score (MASES)** **32**	Evaluates 13 sites, mainly axial and pelvic (e.g. costochondral junctions, iliac crests) Originally for AS, but widely used in PsA Better for axial involvement, less so for peripheral PsA
**SPARCC Enthesitis Index** **33**	Includes 16 sites (axial and peripheral) validated in both axial and peripheral SpA common in clinical trials due to broad coverage and high sensitivity to change
**Ultrasound-based indices (e.g. MASEI)**	Provide objective data on both structural (enthesophytes, erosions, calcifications) and inflammatory (Doppler signal, thickening) changes useful in subclinical or ambiguous cases

In clinical practice, the choice of tool depends on the setting (clinic vs trial), disease phenotype, and the available expertise. A combination of clinical indices and imaging provides the most accurate picture of entheseal involvement and treatment response.

## THERAPEUTIC MANAGEMENT

The management of dactylitis and enthesitis in PsA requires a domain-specific and personalised approach that incorporates both pharmacological and non-pharmacological strategies. These manifestations are markers of more severe disease and have prognostic significance, necessitating early and effective treatment to minimise functional impairment and radiographic progression.

### Non-pharmacological interventions

Non-pharmacological strategies serve as foundational components of comprehensive PsA management. These interventions are especially important in minimising biomechanical stress, improving function, and enhancing adherence to pharmacologic therapy: a) patient education empowers individuals to understand their disease and participate in shared decision-making; b) physical therapy is essential for maintaining range of motion, preventing stiffness, and enhancing overall musculoskeletal function; c) occupational therapy can provide techniques for joint protection and energy conservation; d) custom orthotics and footwear modifications are particularly beneficial in cases of lower limb enthesitis, such as Achilles tendon or plantar fascia involvement; e) weight management reduces axial and entheseal load and has been associated with improved treatment response and reduced inflammatory burden.^34^

### Non-steroidal anti-inflammatory drugs (NSAIDs)

NSAIDs are frequently used as first-line agents for symptomatic relief, particularly in patients presenting with isolated or early dactylitis and enthesitis. They help reduce pain, swelling, and morning stiffness. However, NSAIDs do not modify disease progression and should not be relied upon as monotherapy in active or progressive disease. In addition, gastrointestinal, renal, and cardiovascular risks must be considered, especially in patients with comorbidities or prolonged use.^35^

### Conventional synthetic DMARDs (csDMARDs)

Agents such as methotrexate (MTX), Sulfasalazine (SSZ), and leflunomide (LEF) are often used in PsA patients with predominant peripheral arthritis. However, their efficacy in dactylitis and enthesitis is limited. MTX may offer modest benefit in reducing dactylitic inflammation, particularly when it coexists with polyarthritis, but in a dosage scheme >15mg/w may show better results.^36^ SSZ and LEF show inconsistent and often negligible benefits in enthesitis, and are generally not recommended as monotherapy for these domains. Their role is better justified in mixed phenotypes, especially when systemic inflammation extends beyond the entheses and digits.^37^

### Biologic DMARDs (bDMARDs)

#### Tumour necrosis factor α inhibitors (TNFi)

TNFi including adalimumab, etanercept, infliximab, golimumab, and certolizumab pegol, have demonstrated robust efficacy in both dactylitis and enthesitis. They can reduce inflammation and prevent structural progression, improve imaging and clinical scores, and are considered as first-line biologics for patients with moderate-to-severe PsA with axial, entheseal, and peripheral involvement. In addition, they have an established safety profile and extensive long-term data.^38^

#### Interleukin (IL)-12/23 inhibitors and IL-23 inhibitors

Ustekinumab, targeting the shared p40 subunit of IL-12 and IL-23, is effective across various PsA domains, including skin and joint disease. It can reduce dactylitis and enthesitis burden, and is also particularly useful in patients with comorbid inflammatory bowel disease (IBD) or contraindications to TNFi. While slightly less potent than IL-17 or TNFi in musculoskeletal domains, it offers a favourable safety profile.^35^ Guselkumab and Risankizumab are humanised IL-23 p19 inhibitors approved for moderate-to-severe PsA but they are still lacking robust clinical studies for dactylitis and enthesitis, although there are studies supporting their effectiveness.^39^

#### IL-17 inhibitors

Secukinumab and Ixekizumab, both IL-17A inhibitors as well as Bimekizumab (IL-17A and IL-17F), have shown high efficacy in resolving dactylitis and enthesitis. They are particularly suitable for patients with concurrent axial disease or severe skin involvement. They are also associated with rapid onset of action and sustained improvements in multiple PsA domains.^38^

### Targeted synthetic DMARDs (tsDMARDs)

#### Phosphodiesterase 4 inhibitor (PDE4 inhibitor)

Apremilast, an oral PDE4 inhibitor, offers modest efficacy in dactylitis and enthesitis. It is best suited for patients with mild to moderate PsA, and valuable for patients seeking oral therapies or unable to use biologics due to safety concerns. Its favourable safety profile, with a low immunosuppression risk, makes it a practical option in comorbid populations. However, its clinical effectiveness in severe enthesitis is limited.^40^

#### Janus Kinase (JAK) inhibitors

Tofacitinib and Upadacitinib are JAK inhibitors that disrupt intracellular signalling in pro-inflammatory pathways, including the JAK-STAT axis. They demonstrate broad efficacy across dactylitis, enthesitis, peripheral arthritis, and skin lesions. The also offer oral administration and rapid symptom relief. However, concerns regarding venous thromboembolism (VTE), herpes zoster, and cardiovascular risk warrant careful patient selection and monitoring. JAK inhibitors may be preferred in biologic-experienced patients or those with needle aversion.^41^

## TREATMENT ALGORITHMS AND PERSONALISED MEDICINE

The therapeutic approach to PsA has evolved from a uniform strategy to a personalised, domain-specific model, recognising the clinical heterogeneity of the disease. In this direction, several researcher groups are working in order to find the optimal way of disease management.^42^ One of them, that is usually followed by rheumatologists, is the Group for Research and Assessment of Psoriasis and Psoriatic Arthritis (GRAPPA), which has released treatment recommendation in a domain-specific manner.^43^ Among the musculoskeletal manifestations, dactylitis and enthesitis are considered critical domains that require specific consideration when selecting treatment. These features are associated with more aggressive disease and can independently drive disability and radiographic progression.

Personalised medicine in PsA revolves around selecting therapies based on a) dominant clinical phenotype: dactylitis-predominant, enthesitis-predominant, axial disease; b) presence of poor prognostic markers: early dactylitis, high enthesitis burden, radiographic damage; c) patient-specific factors: such as comorbid conditions (IBD, metabolic syndrome, depression), contraindications, age, fertility considerations, and lifestyle; d) patient preferences regarding route of administration, frequency of dosing, and safety profile; e) prior treatment history and response, including primary failure or secondary loss of efficacy.^44^

This tailored approach maximised therapeutic effectiveness while minimising risks and burden.

### Treatment algorithm for dactylitis and enthesitis (proposed)

We present a proposed treatment algorithm for dactylitis and enthesitis based on disease and patients’ characteristics. An extrapolated algorithm has been chosen as there are not robust head-to-head comparative studies and long-term outcome data for dactylitis and enthesitis in sine.

#### Mild disease

Mild disease refers to non-tender dactylitis, isolated enthesitis, and low disease activity: start with NSAIDs and physical therapy. Consider apremilast for patients seeking oral therapy with lower toxicity risk.

#### Moderate to severe disease

Moderate to severe disease refers to tender dactylitis, multiple entheseal sites, functional limitation, poor prognostic signs: consider early initiation of bDMARDs, especially TNFi or IL-17 inhibitors. TNFi are often first-line due to broad efficacy and long-standing evidence. IL-17 inhibitors are preferred if coexistent axial involvement or significant skin disease is present. IL-12/23 inhibitors may be considered in patients with IBD or TNFi contraindications.

Patients with inadequate response to cs or b DMARDs Escalate to or switch between biologics or ts DMARDs. JAK inhibitors are suitable for refractory cases, provided careful risk assessment is undertaken.

#### Localised symptoms only

Corticosteroid injections to tendon sheaths or entheseal insertions may be used selectively for rapid relief, especially when systemic options are contraindicated or as bridging therapy.

#### Integration of multidomain considerations

The ideal therapeutic choice often addresses multiple disease domains simultaneously. For instance, a patient with enthesitis, psoriasis, and IBD might benefit from Ustekinumab or TNFi. A young active patient with enthesitis and axial symptoms may respond best to IL-17 inhibition, or a patient with needle aversion or poor adherence to injectables may prefer oral agents like apremilast or JAK inhibitors.

#### Role of shared decision-making

Therapeutic success depends not only on efficacy but also on patient adherence and satisfaction. Shared decision-making is essential and should include discussion of expected benefits and risks of each therapy, consideration of patient lifestyle and treatment goals, and use of decision aids and patient-reported outcomes.

## FUTURE DIRECTIONS

Ongoing advances in immunopathogenesis, molecular biology, and imaging technology are rapidly transforming the landscape of PsA management. For the domains of dactylitis and enthesitis, which remain challenging to treat and assess, several exciting future directions are emerging.

### Novel Therapeutic Targets

The therapeutic pipeline in PsA continues to expand with agents targeting previously unexplored cytokine and intracellular signalling pathways: IL-23 inhibitors (guselkumab,^45^ risankizumab,^39^ tildrakizumab^46^) show increasing promise in addressing enthesitis and dactylitis by selectively blocking the p19 subunit of IL-23. These agents may offer improved tolerability and skin-specific efficacy compared to IL-12/23 inhibitors.

TYK2 inhibitors (deucravacitinib) represent a new class of oral agents that modulate cytokine signalling at the intracellular level with greater selectivity than traditional JAK inhibitors. Preliminary studies suggest potential efficacy in peripheral musculoskeletal domains and skin disease with a potentially safer profile.^47^

GM-CSF and other innate immune targets may provide alternative pathways for inflammation resolution, particularly in enthesis-rich microenvironments where traditional adaptive immune modulation is insufficient.^48^

These innovations promise better tolerability, domain-specific efficacy, and options for patients who are refractory to existing biologics.

### Biomarkers and precision medicine

The future of PsA management lies in individualised care guided by biomarkers. Research is ongoing to identify serological, imaging, and genetic biomarkers that can predict the development, severity, and therapeutic response of dactylitis and enthesitis. Biomarkers such as calprotectin, IL-17A/F levels, and T-cell or fibroblast-derived signatures may allow for early stratification and help distinguish between PsA and mimicking conditions such as fibromyalgia. The integration of multi-omics technologies (genomics, proteomics, metabolomics) holds promise in revealing disease-driving mechanisms and informing therapeutic selection.^49^

### Advances in imaging and artificial intelligence

Imaging modalities continue to evolve beyond conventional MSUS and MRI. Whole-body MRI is being refined to quantify systemic enthesitis burden and track inflammation across time points. MSUS elastography may assess biomechanical changes at entheses, detecting early pathological alterations before clinical symptoms arise. Artificial intelligence (AI) and machine learning are being explored to automate enthesitis scoring, increase inter-reader consistency, and identify subclinical changes predictive of disease flare or response.^50^

These technologies will enhance early diagnosis, facilitate remote monitoring, and allow for objective disease quantification.

### Mechanobiology of the enthesis

An emerging frontier in PsA research is the study of mechanobiology – how mechanical forces interact with immune and stromal cells at the enthesis.^51^ Recent studies suggest that mechanical microdamage, particularly in genetically predisposed individuals, may act as a nidus for chronic inflammation via activation of innate immunity. Understanding how mechanotransduction pathways (Piezo channels, integrins, YAP/TAZ signalling) influence inflammatory cascades may uncover new therapeutic targets distinct from traditional cytokine inhibition. Such insights could pave the way for non-immunosuppressive therapies aimed at restoring tissue resilience and modulating local biomechanical stress responses.^52^

### Prevention and early intervention

A future goal is the prevention of PsA onset in patients with psoriasis who demonstrate subclinical entheseal inflammation on imaging. Early identification of “pre-clinical PsA” through risk prediction models, MSUS screening, and biomarker profiling may allow for disease interception with safer early therapies. Trials are underway to test interventions in high-risk psoriasis patients with early entheseal findings, which may significantly reduce the burden of irreversible joint damage. In summary, the future of dactylitis and enthesitis management in PsA lies in the convergence of targeted therapies, precision diagnostics, and a deeper understanding of local tissue biology. These developments promise to shift the paradigm from reactive treatment to proactive, personalised care.

## CONCLUSION

Dactylitis and enthesitis are not only hallmark features of PsA, but also critical indicators of disease severity,^53^ systemic inflammation, and long-term outcomes. Their presence underscores the complex interplay between the skin, joints, entheses, and immune system that defines PsA as a distinct, multifaceted disease within the SpA spectrum.

Clinically, these domains are associated with increased functional impairment,^54^ radiographic progression, and reduced quality of life. Importantly, they often present in early disease, even preceding synovitis, and their timely recognition can facilitate early diagnosis, risk stratification, and initiation of targeted treatment. Their inclusion in classification criteria, disease activity indices, and imaging assessments reflects their centrality in the disease process.

Therapeutically, the past decade has witnessed a paradigm shift in the management of PsA with the development of domain-based treatment algorithms. B- and tsDMARDs have demonstrated significant efficacy in resolving both dactylitis and enthesitis, offering patients the possibility of improved outcomes and remission across multiple domains. Moreover, the growing integration of imaging modalities, particularly high-resolution MSUS and MRI, has enhanced our ability to detect subclinical inflammation and monitor therapeutic response with precision.

Despite these advances, several challenges remain. A proportion of patients continue to experience refractory dactylitis or enthesitis despite optimal therapy. Variability in clinical assessment tools and the overlap of entheseal pain with non-inflammatory conditions such as fibromyalgia can complicate evaluation. Additionally, real-world data on long-term outcomes and the relative effectiveness of newer agents in these specific domains are still evolving.

Looking forward, emerging therapies targeting novel pathways such as TYK2, as well as mechanobiology-driven interventions, hold promise for addressing treatment gaps. The integration of biomarkers, machine learning, and patient-reported outcomes into clinical practice will further refine our approach, moving toward truly personalised, precision medicine in PsA. In summary, dactylitis and enthesitis are not peripheral features, they are core disease domains that demand early recognition, validated assessment, and individualised, mechanism-based treatment. Continued research and clinical innovation will be essential in improving care for patients living with PsA.

This review aimed to synthesise the current evidence on the therapeutic approaches and clinical relevance of enthesitis and dactylitis in PsA. In conclusion, it offers the reader an integrated and comprehensive perspective on all facets of these important clinical manifestations.

## References

[1] Pelechas E Kaltsonoudis E Migkos MP Koletsos N Karagianni PG Drosos AA State of the art review on the treatment of psoriatic disease. Mediterr J Rheumatol 2024;35:66–72. doi: 10.31138/mjr.040123.sot 38736956 PMC11082764

[2] Kaltsonoudis E Karagianni P Memi T Pelechas E. State-of-the-art review on the treatment of Axial Spondyloarthritis. Med Sci (Basel) 2025;13:32. doi: 10.3390/medsci13010032 40137452 PMC11944150

[3] Pelechas E Kaltsonoudis E Voulgari PV Drosos AA. Psoriatic Arthritis. In: Illustrated handbook of Rheumatic and Musculo-Skeletal diseases. Springer, Cham 2023, pp 195–225. doi: 10.1007/978-3-031-47379-1_9

[4] Fragoulis GE Papagoras C Gazi S Mole E Krikelis M Voulgari PV Disease profile and achievement of therapeutic goals in a modern, nationwide cohort of 923 patients with psoriatic arthritis. Mediterr J Rheumatol 2023;34:418–26. doi: 10.31138/mjr.301223.dpa 38282940 PMC10815515

[5] Bagel J Schwartzman S. Enthesitis and Dactylitis in Psoriatic Disease: A guide to for dermatologists. Am J Clin Dermatol 2018;19:8390852. doi: 10.1007/s40257-018-0377-2 PMC6267546 30117018

[6] Smith JA Colbert RA. The IL-23/IL-17 axis in Spondyloarthritis pathogenesis: Th17 and beyond. Arthritis Rheumatol 2014;66:231–41. doi: 10.1002/art.38291 24504793 PMC4058712

[7] Gialouri CG Evangelatos G Fragoulis GE. Choosing the appropriate target for the treatment of psoriatic arthritis: TNFa, IL-17, IL-23 or JAK inhibitors? Mediterr J Rheumatol 2022;33(Suppl 1):140–161. Doi: 10.31138/mjr.33.1.150. PMC9450184 36127928

[8] Gasparyan AY Ayvazyan L Blackmore H Kitas GD. Writing a narrative biomedical review: considerations for authors, peer reviewers, and editors. Rheumatol Int 2011;31(11):1409–17. Doi: 10.1007/s00296-011-1999-3 21800117

[9] Pelechas E Kaltsonoudis E Voulgari PV Drosos AA. Psoriatic Arthritis. In; Illustrated handbook of rheumatic and musculoskeletal diseases. Springer, Cham, 2019, pp 93–119. doi: 10.1007/978-3-030-03664-5_5

[10] Yamamoto T. Angiogenic and inflammatory properties of psoriatic arthritis. ISRN Dermatol 2013;2013:630620. Doi: 10.1155/2013/630620 23819059 PMC3683428

[11] McGonagle D Tan AL Watad A Helliwell P. Pathophysiology, assessment and treatment of psoriatic dactylitis. Nat Rev Rheumatol 2019;15:113–22. doi: 10.1038/s41584-018-0147-9 30610219

[12] Fragoulis GE Siebert S. The role of IL-23 and the use of IL-23 inhibitors in psoriatic arthritis. Musculoskeletal Care 2022;20:S12–S21. doi: 10.1002/msc.1694 36069174 PMC9825973

[13] Kavvadias A Karavasili M Pelechas E Christodoulou V Voulgari PV Katsanos KH Rheumatic manifestations in patients with idiopathic inflammatory bowel disease: a single tertiary centre, interdisciplinary study. Mediterr J Rheumatol 2025;36:215–9. doi: 10.31138/mjr.291123.ept 40757119 PMC12312475

[14] Russell T Bridgewood C Rowe H Altaie A Jones E McGonagle D. Cytokine “fine tuning” of enthesis tissue homeostasis as a pointer to spondyloarthritis pathogenesis with a focus on relevant TNF and IL-17 targeted therapies. Semin Immunopathol 2021;43:193–206. Doi: 10.1007/s00281-021-00836-1 33544244 PMC7990848

[15] Araujo EG Schett G. Enthesitis in psoriatic arthritis (Part 1): pathophysiology. Rheumatology (Oxford) 2020;59:i10–i14. doi: 10.1093/rheumatology/keaa039 32159793 PMC7065460

[16] Pelechas E Kaltsonoudis E Voulgari PV Drosos AA. Musculoskeletal ultrasound In rheumatology. In: illustrated handbook of rheumatic and musculoskeletal diseases.Springer, Cham, 2023. doi: 10.1007/978-3-031-47379-1_4

[17] Taylor W Gladman D Helliwell P Marchesoni A Mease P Mielants H CASPAR Study Group. Classification criteria for psoriatic arthritis: development of new criteria from a large international study. Arthritis Rheum 2006;54:2665–73. doi: 10.1002/art.21972 16871531

[18] Urruticoechea-Arana A Moreno M Pujol M Clavaguera T. Ultrasound in the evaluation of dactylitis and enthesitis in psoriatic arthritis. Eur J Rheumatol 2024;11:S298–S304. doi: 10.5152/eurjrheum.2024.21096 39311552 PMC11459574

[19] Tan AL Fukuba E Halliday NA Tanner SF Emery P McGonagle D. High-resolution MRI assessment of dactylitis in psoriatic arthritis shows flexor tendon pulley and sheath-related enthesitis. Ann Rheum Dis 2015;74:185–9. doi: 10.1136/annrheumdis-2014-205839 25261575 PMC4283670

[20] Dubash S Alabas OA Michelena X Garcia-Montoya L Wakefield RJ Helliwell PS Dactylitis is an indicator of a more severe phenotype independently associated with greater SJC, CRP, ultrasound synovitis and erosive damage in DMARD-naïve early psoriatic arthritis. Ann Rheum Dis 2021;81:490–5. doi: 10.1136/annrheumdis-2021-220964 34893470 PMC8921567

[21] Yang F Lu C Liu H Dou L Wand Y Li H. Enthesitis in patients with psoriatic arthritis: a nationwide data from the Chinese registry of psoriatic arthritis (CREPAR). Chin Med J (Engl) 2023;136:951–8. doi: 10.1097/CM9.0000000000002646 37036901 PMC10278716

[22] Mease PJ Liu M Rebello S Hua W McLean RR Hur P Disease characteristics, quality of life, and work productivity by enthesitis site: real-world data from the US Corrona Psoriatic Arthritis/Spondyloarthritis Registry. J Rheumatol 2021;48:367–375. doi: 10.3899/jrheum.191117 32482647

[23] Coates LC Orbai A-M Azevedo VF Cappelleri JC Steinberg K Lippe R Results of a global, patient-based survey assessing the impact of psoriatic arthritis discussed in the context of the psoriatic arthritis impact of disease (PsAID) questionnaire. Health Qual Life Outcomes 2020;18:173. doi: 10.1186/s12955-020-01422-z 32513190 PMC7282161

[24] Sakellariou G Girolimetto N Tinazzi I Canzoni M Filippou G Batticciotto A The ultrasonographic spectrum of toe dactylitis in psoriatic arthritis: a descriptive analysis. Clin Rheumatol 2025;44:1939–47. Doi: 10.1007/s10067-025-07395-y 40111541 PMC12078354

[25] Girolimetto N Macchioni P Possemato N Tinazzi I Bascherini V Citriniti G Musculoskeletal ultrasound in monitoring clinical response to treatment in acute symptomatic psoriatic dactylitis: results from a multicenter prospective observational study. J Clin Med 2020;9:3127. doi: 10.3390/jcm9103127 32992634 PMC7601129

[26] Bakewell CJ Olivieri I Aydin SZ Dejaco C Ikeda K Gutierrez M Ultrasound and magnetic resonance imaging in the evaluation of psoriatic dactylitis: status and perspectives. J Rheumatol 2013;40:1951–7. doi: 10.3899/jrheum.130643 24187105

[27] Filippucci E Smerilli G Di Matteo A Grassi W. Ultrasound definition of enthesitis in spondyloarthritis and psoriatic arthritis: arrival or starting point? Ann Rheum Dis 2021;80:1373–5. doi: 10.1126/annrheumdis-2021-220478 34172503

[28] Macía-Villa C De Miguel E. Updating the use of the Madrid Sonographic Enthesis Index (MASEI): a systematic review of the literature. Rheumatology (Oxford) 2020;59:1031–40. doi: 10.1093/rheumatology/kez356 31750519

[29] Balint PV Kane D Wilson H McInnes IB Sturrock RD. Ultrasonography of entheseal insertions in the lower limb in spondyloarthropathy. Ann Rheum Dis 2002;61:905–10. doi: 10.1136/ard.61.10.905 12228161 PMC1753913

[30] Helliwell PS Firth J Ibrahim GH Melsom RD Shah I Turner DE. Development of an assessment tool for dactylitis in patients with psoriatic arthritis. J Rheumatol 2005;32:1745–50. 16142872

[31] Healy PJ Helliwell PS. Measuring clinical enthesitis in psoriatic arthritis: assessment of existing measures and development of an instrument specific to psoriatic arthritis. Arthritis Rheum 2008;59:686–91. doi: 10.1002/art.23568 18438903

[32] Heuft-Dorennbosch L Spoorenberg A van Tubergen A Landewe R van der Tempel H Mielants H Assessment of enthesitis in ankylosing spondylitis. Ann Rheum Dis 2003;62:127–132. doi: 10.1136/ard.62.2.127 12525381 PMC1754445

[33] Maksymowych WP Mallon C Morrow S Shojania K Olszynski WP Wong RL Development and validation of the spondyloarthritis research consortium of Canada (SPARCC) enthesitis index. Ann Rheum Dis 2009;68:948–53. doi: 10.1136/ard.2007.084244 18524792

[34] Perrotta FM Scriffignano S Benfaremo D Ronga M Luchetti MM Lubrano E. New insights in physical therapy and rehabilitation in psoriatic arthritis: a review. Rheumatol Ther 2021;8:639–49. doi: 10.1007/s40744-021-00298-9 33710586 PMC8217348

[35] Manara M Carriero A Congia M Galluzzo C Pandolfi M Salvato M Non-steroidal anti-inflammatory drugs in psoriatic arthritis: clinical practice suggestions based on scientific evidence and expert opinion. Clin Exp Rheumatol 2025;43:897–906. doi: 10.55563/clinexprheumatol/j7b6t5 40153331

[36] Appani KS Devarasetti PK Irlapati RVP Rajasekhar L. Methotrexate achieves major cDAPSA response, and improvement in dactylitis and functional status in psoriatic arthritis. Rheumatology (Oxford) 2019;58:869–73. 30590763 10.1093/rheumatology/key369

[37] Mathew AJ Sutton M Pereira D Gladman DD Chandran V. Effectiveness of disease-modifying antirheumatic drugs for enthesitis in a prospective longitudinal psoriatic arthritis cohort. J Rheumatol 2022;49:1020–5. doi: 10.3899/jrheum.211231 35649547

[38] Mourad A Gniadecki R. Treatment of dactylitis and enthesitis in psoriatic arthritis with biologica agents: a systematic review and metaanalysis. J Rheumatol 2020;47:59–65. doi: 10.3899/jrheum.180797 30824641

[39] Kwatra S Khattri S Amin AZ Ranza R Kaplan B Shi L Enthesitis and dactylitis resolution with risankizumab for active psoriatic arthritis: integrated analysis of the randomized KEEPsAKE 1 and 2 trials. Dermatol Ther (Heidelb) 2024;14:1517–30. doi: 10.1007/s13555-024-01174-4 38739215 PMC11169338

[40] Gullo AL Becciolini A Parisi S Del Medico P Farina A Visalli E Therapeutic effects of apremilast on enthesitis and dactylitis in real clinical setting: an Italian multicenter study. J Clin Med 2023;12:3892. doi: 10.3390/jcm12123892 37373587 PMC10299365

[41] Harkins P Burke E Swales C Silman A Conway R. Are Janus kinase inhibitors safe and effective in treating the key clinical domains of psoriatic arthritis? A systematic review and meta-analysis. Int J Rheum Dis 2022;26:31–42. doi: 10.1111/1756-185X.14447 36184741 PMC10092437

[42] Al-Homood IA Ghanim NA Fatani MIA Hussein AH Alolaiwi AM Abualiat A The Saudi consensus recommendations for the management of psoriatic arthritis (2023). Clin Rheumatol 2024;43:879–94. Doi: 10.1007/s10067-024-06867-x 38217738 PMC10876726

[43] Coates LC Soriano ER Corp N Bertheussen H Duffin KC Campanholo CB Group for Research and Assessment of Psoriasis and Psoriatic Arthritis (GRAPPA): updated treatment recommendations for psoriatic arthritis 2021. Nat Rev Rheumatol 2022;18:734. doi: 10.1038/s41584-022-00861-w PMC9828276 36216924

[44] Gunawardana S Tillet W Coates LC. Achieving precision medicine in psoriatic arthritis: novel treatment strategies and trial designs. Rheum Dis Clin North Am 2025;51:495–509. doi: 10.1016/j.rdc.2025.05.007 40681284

[45] Rahman P McInnes IB Deodhar A Schett G Mease PJ Shawi M Association between enthesitis/dactylitis resolution and patient-reporteed outcomes in guselkumab-treated patients with psoriatic arthritis. Clin Rheumatol 2024;43:1591–604. doi: 10.1007/s10067-024-06921-8 38472528 PMC11018666

[46] Mease PJ Chohan S Fructuoso FJG Luggen ME Rahman P Raychaudhuri SP Efficacy and safety of tildrakizumab in patients with active psoriatic arthritis: results of a randomised, double-blind, placebo-controlled, multiple-dose, 52-week phase IIb study. Ann Rheum Dis 2021;80:1147–57. doi: 10.1136/annrheumdis-2020-219014 33985942 PMC8372392

[47] Martins A Lé AM Torres T. Deucravacitinib for the treatment of psoriatic arthritis: the evidence so far. Drugs Context 2023;12:2023-2-7. doi: 10.7573/dic.2023-2-7 PMC10166261 37168876

[48] Fuentelsaz-Romero S Cuervo A Estrada-Capetillo L Celis R García-Campos R Ramírez J GM-CSF expression and macrophage polarization in joints of undifferentiated arthritis patients evolving to rheumatoid arthritis or psoriatic arthritis. Front Immunol 2021;11:613975. doi: 10.3389/fimmu.2020.613975 33679701 PMC7925849

[49] Cai YX Zheng DS Chen XL Bai ZP Zhang J Deng W An integrated multi-omics analysis identifies protein biomarkers and potential drug targets for psoriatic arthritis. Commun Biol 2025;8:240. doi: 10.1038/s42003-025-07698-5 39953266 PMC11828935

[50] Pournara E Kormaksson M Nash P Ritchlin CT Kirkham BW Ligozio G Clinically relevant patient clusters identified by machine learning from the clinical development programme of secukinumab in psoriatic arthritis. RMD Open 2021;7:e001845. doi: 10.1136/rmdopen-2021-001845 34795065 PMC8603280

[51] Killian ML. Growth and mechanobiology of the tendon-bone enthesis. Semin Cell Dev Biol 2021;123:64–73. doi: 10.1016/j.semcdb.2021.07.015 34362655 PMC8810906

[52] McGonagle D Tan AL. The enthesis in psoriatic arthritis. Clin Exp Rheumatol 2015;33:S369. 26472070

[53] Grazio S Šitum M Grubišić F Kavanagh HS Vajdić ID Krstanović K Association of enthesitis with severity of psoriasis in psoriatic arthritis: an observational study. Rheumatol Int 2024;44:2891–6. Doi: 10.1007/s00296-024-05730-1 39402163

[54] Patience A Steultjens M Siebert S Hendry G. Significant functional impairment and disability in individuals with psoriatic arthritis and Achilles tendon pain: a cross-sectional observational study. Rheumatol Int 2024;44:1469–79. Doi: 10.1007/s00296-024-05629-x 38850322 PMC11222213

